# Second malignant neoplasms in lymphomas, secondary lymphomas and lymphomas in metabolic disorders/diseases

**DOI:** 10.1186/s13578-022-00763-0

**Published:** 2022-03-12

**Authors:** Youxi Yu, Xiaoju Shi, Xingtong Wang, Ping Zhang, Ou Bai, Yan Li

**Affiliations:** 1 Department of Hepatobiliary and Pancreatic Surgery, The First Hospital of Jilin University, No. 71. Xinmin Street, Changchun, 130021 Jilin China; 2 Department of Hematology, Cancer Center, The First Hospital of Jilin University, No. 71. Xinmin Street, Changchun, 130021 Jilin China; 3 Division of Surgical Oncology, Department of Surgery, University of Louisville School of Medicine, 511 S Floyd ST MDR Bldg Rm324, Louisville, KY 40202 USA

**Keywords:** Lymphoma, Lymphomagenesis, Second malignant neoplasm, Secondary lymphoma, Solid tumor, Metabolic diseases

## Abstract

With inconsistent findings, evidence has been obtained in recent years that metabolic disorders are closely associated with the development of lymphomas. Studies and multiple analyses have been published also indicating that some solid tumor survivors develop a secondary lymphoma, whereas some lymphoma survivors subsequently develop a second malignant neoplasm (SMN), particularly solid tumors. An interaction between the multiple etiologic factors such as genetic factors and late effects of cancer therapy may play an important role contributing to the carcinogenesis in patients with metabolic diseases or with a primary cancer. In this review, we summarize the current knowledge of the multiple etiologic factors for lymphomagenesis, focusing on the SMN in lymphoma, secondary lymphomas in primary cancers, and the lymphomas associated to metabolic disorders/diseases, which have been received less attention previously. Further, we also review the data of coexistence of lymphomas and hepatocellular carcinoma (HCC) in patients with infection of hepatitis C virus and hepatitis B virus.

**Graphical Abstract:**

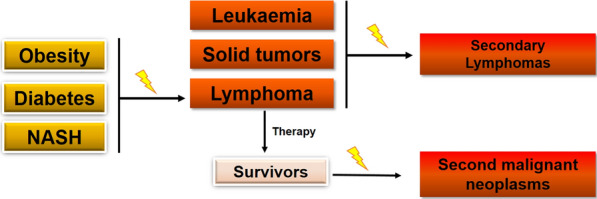

## Introduction

Based on morphology, immunophenotype, genetic alterations, and clinical features, lymphomas have been categorized into more than 80 distinct entities, principally according to the World Health Organization classification [1]. In fact, lymphomas are a heterogeneous group of hematological malignancies and multifactorial etiopathogenesis has been proposed for the development of lymphomas. Lymphoma therapy is varying greatly, based on type of lymphoma, stage, histopathological features, and patient’s factors such as age and symptoms. The most common treatment includes chemotherapy, radiotherapy, immunotherapy, cellular therapy, and in rare cases, surgery. The published studies indicate that some lymphoma survivors subsequently develop a second malignant neoplasm (SMN), particularly solid tumors, whereas some solid tumor survivors develop a secondary lymphoma. Over the years, the disease-related etiologies have been extrapolated from current literature for those patients with metabolic diseases who may be at high risk for lymphoma development. While appropriate clinical manipulation for cancer survivors and patients with metabolic diseases may prevent or even reverse the lymphomagenetic process, it is really important to gain insight into the lymphomagenesis for those patients but received less attention. In this review article, PubMed was searched from inception to 2021 to discuss the available reports on either lymphoma patients subsequently being diagnosed with SMNs or development of secondary lymphomas from the patients with primary cancers, and the association between lymphomas and metabolic diseases. A summary of studies reported in the literature is presented by lymphoma type and subtype, including classical Hodgkin lymphoma (HL), EBV‐Hodgkin lymphoma, non-Hodgkin lymphoma (NHL) including Diffuse large B-cell lymphoma (DLBCL), follicular lymphoma (FL), mantle cell lymphoma (MCL), primary effusion lymphoma (PEL), marginal zone lymphoma (MZL), and T‐cell lymphoma.

## Lymphomas and second malignant neoplasms

Over the past decades, treatment of lymphoma in adults and children has become quite successful because of the improved therapeutic radiation techniques [2, 3] and the introduction of effective combination chemotherapeutic regimens [4, 5]. However, it has been noticed a substantial increase of SMN in lymphoma survivors with a longer life span. As childhood cancer survivors have a longer life expectancy than adult cancer survivors, SMN is considered as one of the most devastating late effects of childhood cancers which call attention world-widely because the occurrence of SMNs in childhood cancer survivors shortens their life expectancy and influences their quality of life [6–10]. In a cohort of children and adolescents being treated between 1960 and 1989 for Hodgkin’s disease, cumulative percentage of patients who developed a SMN after diagnosis was 3.82% ± 1.53% at 10 years; 8.02% ± 2.35% at 15 years; 12.71% ± 3.20% at 20 years; 16.11% ± 3.88% at 25 years; and 26.27% ± 6.75% at 30 years, respectively [11]. Recently, a larger cohort of children and adolescents with SMNs after childhood NHL therapy (n = 189) was reported, with the details of 5 major categories of SMNs (myeloid SMNs, lymphoid SMNs, carcinomas, central nervous system (CNS) tumors, “other” SMNs) as well as the associated NHL subtypes [12]. In this study, a higher risk for the patients with lymphoblastic lymphomas to develop an SMN is suggested, while the patients with lymphoid SMNs, carcinomas, and “other” SMNs showed a better outcome than the patients with myeloid SMNs and CNS tumors (p < 0.0001) [12]. Besides the childhood cancer survivors, more adults after a diagnosis of cancer are living longer than ever before. Now, the cancer survivors account for around 5% of the US population, from 3 million in 1971 to more than 16.9 million in 2019, and this number is expected to continue growing to reach 22.1 million by 2030 [13]. The increased lifetime could also result in an increased risk of SMNs in adults, most importantly, the patients who developed secondary malignancies often have a poor prognosis. In a cohort of 6171 NHL patients being identified within the tumor registries in Sweden, Ontario, and Iowa and the Netherlands Cancer Institute, SMNs were reported in 541 subjects [observed-to-expected ratio (O/E) = 1.37; 95% CI = 1.26–1.49], with actuarial risk of 21% for developing a SMN at 3–20 years after diagnosis of NHL, compared to the population expected cumulative risk of 15% [14]. In a cohort of 592 patients being diagnosed as NHL at Osaka Medical Center for Cancer and Cardiovascular Diseases, significantly increased risk was found for HCC (O/E = 4.36, 95% CI = 1.99–8.28; O = 9) and non-lymphocytic leukemia (O/E = 26.17, 95% CI = 5.26–76.46; O = 3). The patients who received chemotherapy as the NHL treatment had a significantly increased risk of HCC (O/E = 5.91, 95% CI = 2.70–11.23; O = 9), in which 8 out of 9 patients showed evidence of cirrhosis at the time of HCC diagnosis [15]. In a cohort of 2,456 patients with NHL who were first treated from 1973 to 2000 from centers in the British National Lymphoma Investigation, the relative risks (RRs) were significantly elevated for all malignancies combined (RR = 1.3; 95% CI, 1.1 to 1.6), for leukemia (RR = 8.8; 95% CI, 5.1 to 14.1) and lung cancer (RR = 1.6; 95% CI, 1.1 to 2.3), compared with expectations based on general population cancer rates [16]. In a larger cohort of 109,451 NHL first primary cases, a significant (P < 0.001) increase was observed for 18 various cancers, including lip, tongue, oropharynx, stomach, small intestine, colon, liver, nose and nasal cavity, lung, soft tissue, skin melanoma and nonmelanoma skin cancer, bladder, kidney, thyroid, Hodgkin’s lymphoma, lymphoid leukaemia and myeloid leukaemia [17]. The overall increase in the risk of a SMN was 47%, while the RR was higher along with the increasing time of follow-up, being 1.37 (95% CI 1.32–1.43) in the 1–4-year follow-up period, and 1.67 (95% CI 1.59–1.76) after 10 years or more [17]. In 2015, a cohort study of 2548 Hodgkin’s lymphoma patients from the German, Austrian, and Swiss with follow up over 30 years reported that the cumulative incidence of SMN at 20, 25, and 30 years was 7%, 11.2%, and 18.7%, respectively [18]. For all types of SMN, the standardized incidence rate (SIR) was 9.1 and the absolute excess risk (AER) was 16.8. Of note, 85% (105 out of 123) patients with SMNs had a tumor in the irradiated region, implying the risk of late sequelae of radiotherapy in HL patients for development of SMN [18].

It is generally accepted that the occurrence of SMN is attributed mostly to genetic factors, environmental factors, and late effects of cancer therapy (particularly chemo- and radiotherapy for the first primaries) [19]. The genetic factors played an important role in SMNs which was supported by the finding of an increased risk of SMNs among lymphoma patients (HL = 7,476; NHL = 25,941) with a family history of cancer, defined as a diagnosis of any cancer in the first‐degree relatives [20]. Compared to HL patients with a negative family history of cancer, a significantly increased risk of breast cancer (RR = 1.81, 95% CI: 1.04–3.16) was found among the HL patients with a positive family history of cancer, while increases of non-melanoma skin cancer (RR = 1.94, 95% CI: 0.86–4.38) and lung cancer (RR = 1.99, 95% CI; 0.73–5.39) were also observed even though the statistical significance was not reached [20]. Heritable diseases and acquiring gene mutations of malignant cells have been suggested as the potential genetic mechanism of SMN. For example, retinoblastoma is frequently caused by inherited mutations of the retinoblastoma gene (RB1), a tumor suppressor gene. In cohort studies of heritable retinoblastomas, the estimated cumulative risk of second bone cancer by 20 years from 3-year survival was 6.0% based on a mean follow-up period of 13.7 years [21] and 7.2% based on a mean follow-up period of 17.6 years [22], respectively. In a retrospective study of retinoblastoma patients, the cumulative risk of second cancer within 20 years of bilateral or heritable retinoblastoma clustered was around 10%, and the predominant types of tumors observed were bone and soft-tissue sarcomas [23]. Other susceptibility genes and associated diseases such as NF1 gene (Recklinghausen's disease), *WT1* gene (Wilms' tumour), gene *APC* (adenomatous polyposis coli), and ATM gene (ataxia telangiectasia) could also contribute to SMNs in the patients with family histories [24]. As only limited studies focus on the genetic factors of SMNs in lymphomas, further study is needed to study the genetic factors of SMN, in particularly to investigate the heritable diseases and associated genes to elucidate the potential mechanism(s) of SMN carcinogenesis in lymphoma conditions.

Environmental and occupational factors are also associated with an increased risk of lymphomas [25, 26]. Studies indicated that there is a high risk of NHL associated to environmental/occupational exposures, with either historically exposed chemicals or specific groups of chemicals such as pesticides and herbicides. Some environmental/occupational exposed chemicals such as insecticides could apply to all the NHL subtypes, while others might be for specific lymphoma subtypes only, e.g., benzene applied to follicular lymphoma [26, 27]. Of note, selection of pesticide chemical types should be considered carefully, e.g., it was found an elevated risk in NHL among the farmers who reported ever being exposed to terbufos, but a decreased risk with organochlorine insecticides and phenoxy herbicides, based on a meta-analysis of farmers from three cohort studies conducted by Consortium of Agricultural Cohort Studies (AGRICOH) [28]. Interestingly, the risk of myeloid malignancies decreased with specific animal species e.g., pig farming where the individuals were exposed to high concentrations of bioaerosols like organic dust in addition to pesticides, however increased risk of myeloproliferative neoplasms was seen with increasing number of sheep/goats [29]. In addition to the farming occupation, the increased risk of NHL subtypes is also associated with the industrial pollutants and productions, including polychlorinated biphenyl [30], trichloroethylene [31], hair dye [32], etc. Several review articles have contributed greatly to the current knowledge on the roles of environmental exposures to the etiology and molecular pathogenesis in lymphomas as well as lymphoma subtypes [33–35]. Environmental/occupational determinants have been associated with increased SMNs [19], however the analytic studies in terms of increased risk of SMNs in a cohort of lymphoma patients are not available yet. Further large-scale studies on environmental epidemiology and basic science research to elucidate the molecular mechanisms of chemical exposures on the lymphomagenesis-associated SMNs are urgently.

**Fig. 1:**
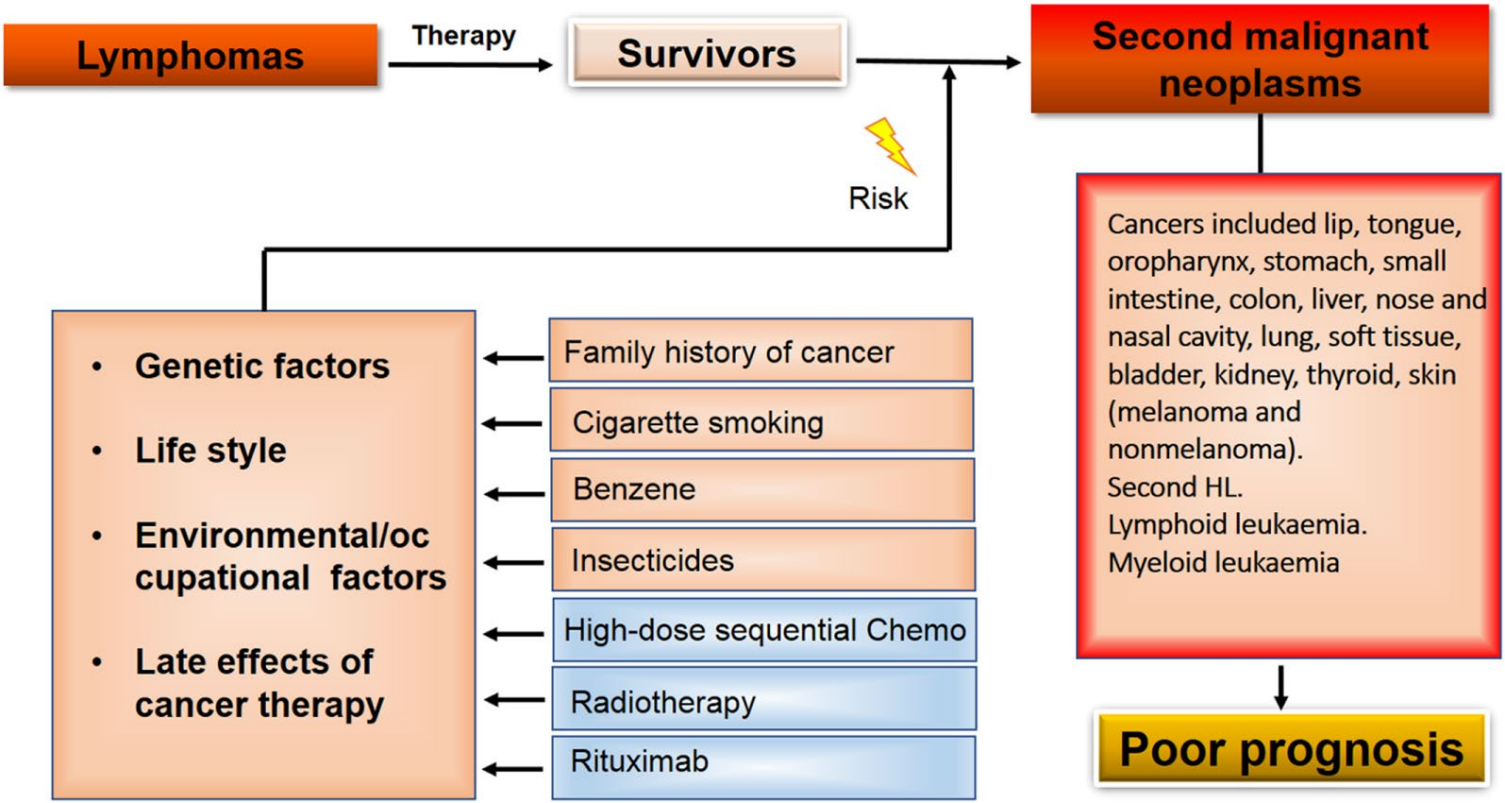
Schematic diagram for risk of second malignant neoplasm in lymphomas and associated risk factors

The late effects of lymphoma therapy in regard to the increased risk of SMNs have been well-studied. Based on individual patient data (IPD) from patients treated for newly diagnosed HL, a meta-analysis was performed to investigate the possible changes in the risk of SMNs [36]. It was found that consolidating radiotherapy was associated with an increased rate of SMNs, while optimizations of treatments such as fewer chemotherapy cycles and reduction of the radiation field/dose did not markedly affect the SMNs [36]. The risk of secondary acute myeloid leukaemia and myelodysplastic syndrome (AML/MDS) was also increased even though efficacy was improved among patients treated with intensified chemotherapy protocols, suggesting the importance to make treatment decisions which could be tailored for individual patients [36]. In a 20-year retrospective follow-up study, total of 1,347 lymphoma patients being treated with a high-dose sequential (HDS) program were analyzed for the cumulative incidence of SMNs [37]. The results showed that the cumulative incidence of secondary myelodysplasia/acute leukemia (sMDS/AL) was 3.09% for 5 years and 4.52% for 10 years. In addition to the secondary hematologic malignancy, the development of secondary solid tumors in lymphoma patients was called attention, especially in the patients treated with monoclonal antibody rituximab which was reported as an independent risk factor for solid tumor development and the cumulative incidence of solid tumors was 2.54% for 5 years and 6.79% for 10 years, respectively [37]. In a study of HL survivors being treated with abdominal radiotherapy or procarbazine-containing chemotherapy, a fivefold increased risk of developing colorectal cancer (CRC) was not only related to therapy but also related to a somatic gene mutation such as the mutations of mismatch repair genes [38]. Haematopoietic stem-cell transplantation (HSCT) was wildly reported for the patients at increased risk of developing SMN [39–45], i.e., one study reporting an RR of 12.0 (95% CI 8.2–14.5) [46]. The literature about the HSCT-related SMNs has been reviewed in detail recently [47]. In lymphoma survivors, solid cancers including breast cancer, thyroid cancer, lung cancer, gastrointestinal cancers, and sarcomas [48, 49] account for the majority of SMNs, while the most common SMN in HL survivors is secondary breast cancer [50, 51]. Accumulating evidence suggests that the therapy-related risk of SMNs increases after a delay of about 10 to 15 years and depends on age at treatment, patient characteristics, differences in radiotherapy and chemotherapy, radiation dose, and length of follow-up [52]. In HL patients, the age at treatment is the most important risk modifying factor for secondary breast cancer development, the younger the patients are, the higher the risk is [53]. The excess risk in younger patients could be related to the increased sensitivity of the mammary gland to the radiation-effect and it was supported by a study in which women (aged ≤ 35) treated with supra-diaphragmatic radiotherapy for HL at high risk when they received radiotherapy closer to the menarche [54]. Radiotherapy field size is also an important issue, lower risk of secondary breast cancer was found in women who received supra-diaphragmatic field radiotherapy not including the axilla compared to those treated with complete mantle-field radiotherapy [55]. Another important issue needs to pay attention to when assessing the risk of SMNs is the dose distribution variations of therapeutic radiation. While high-doses, intermediate-doses, and low-doses radiotherapy showed inconsistent results, assessing the doses being delivered should consider multiple variable parameters such as patient height, weight, size, volume and shape of the tumor, irradiation modalities, etc. The inconsistent results were also found in the chemotherapy when assessing the risk of SMNs because chemotherapy regimens could not be as a sole modality for lymphoma therapy [50, 51]. Likewise, multiple variable parameters as aforementioned should considered to assess the late effects of chemotherapy in lymphoma patients. The risk of second malignant neoplasm in lymphomas and associated risk factors are summarized in Fig. 1.

## Secondary lymphomas

As improvements in survival of solid tumors also influence the potential risk of development of hematological malignancies, it is of interest to note whether there is a higher risk of lymphoma in the patients previously diagnosed with solid tumors than the general population. Previously, a study was performed to determine the risk of the secondary NHL in a cohort of 5484 consecutive children with a variety of malignancies during a period of 27 years [56]. The probability of secondary NHL in this cohort after the diagnosis of the first malignancy was 0.05% (95% CI, 0.01%, 0.2%) at 5–10 years and 0.16% at 15 years (0.04%, 0.63%). Twenty-four children with secondary NHL had initial lymphohematopoietic neoplasms including Hodgkin's disease (n = 18), acute lymphoblastic leukemia (n = 4), and acute myelogenous leukemia (n = 1), while one child had astrocytoma [56]. In Japan, a retrospective cohort study of 2786 patients who were diagnosed with breast cancer between 1970‐1995 (average follow‐up period, 8.6 years) showed that the subjects aged 20–49 years at the time of diagnosis of breast cancer had a significantly increased risk of NHL (O/E = 6.3, 95% CI = 1.7–16.1)[57]. A population-based cohort of 288,390 colorectal cancer (CRC) patients diagnosed between 1973 and 2012 from the database (Surveillance, Epidemiology, and End Results) was retrospectively reviewed to estimate the relative risk for subsequent primary malignancies [58]. In comparison with the general population, CRC patients showed an significantly increased risk of secondary lymphoma (standardized incidence ratios: 0.92) as well as other secondary solid tumors [58]. A study evaluated the occurrence of renal cell cancer (RCC) and hematologic malignancies in 700 individual patients associated with more than 700 pedigrees of families [59]. The results showed a personal history of both RCC and hematologic malignancies for 26 patients in which 13 NHL patients and 4 HL patients were identified. Interestingly, 74 patients with RCC were noted to have 95 family members with hematologic malignancies in which 42 NHL and 12 HL were identified [59]. In China, a cohort of 836 DLBCL patients between 2013 and 2018 was retrospectively studied to investigate the personal history of solid tumors. Total 34 patients were found to have both DLBCL and solid tumors (including liver cancer, stomach cancer, lung cancer, prostate cancer, breast cancer, CRC, renal cell cancer, cervical cancer, ovarian cancer, thyroid cancer, and pancreatic cancer). Among the 34 patients, 30 patients (88%) were diagnosed with solid tumors before DLBCL diagnosis, while 4 patients (12%) were diagnosed simultaneously for a solid tumor and DLBCL [60]. The cohort studies of secondary lymphomas are summarized in Table 1. A secondary lymphoma in HCC patients was also observed [61, 62], however the retrospective cohort study and systematic analysis in regard to the substantial lymphomas in HCC patients were not available. Whereas the etiologic factors such as hepatitis virus infection are associated with both HCC and lymphomas [63–66], the additional study, therefore, is needed to clarify the association between HCC and secondary lymphomas to enhance the understanding of the oncogenic potential of lymphomas in patients with primary HCC.

**Table 1:** Secondary lymphomas

Study center	Year	Primary malignancies	Cases	Secondary lymphomas	Cases	Reference
St. Jude Children’s Research Hospital and the University of Tennessee	1962–1989	HL; Acute lymphoblastic leukemia; Astrocytoma; Other malignancies	5484	NHL	24	35
Osaka Medical Center for Cancer and Cardiovascular Diseases	1970–1994	Breast carcinoma	2786	HL	7	36
The National Cancer Institute’s Surveillance, Epidemiology, and End Results (SEER) program database, nine registries including Atlanta, Connecticut, Detroit, Hawaii, Iowa, New Mexico, San Francisco–Oakland, Seattle–Puget Sound, and Utah	1973–2012	Colorectal cancer	233,890	lymphoma	lymphoma (SIR 0.92) compared with the general population	37
Lady of Mercy Cancer Center	1996–2016	Renal cell cancer; Hematologic malignancies	294	NHL; HL	HL: 17 NHL: 184	38
Department of Hematology at Ruijin Hospital	2013–2018	History of solid tumors: Gastric cancer; Renal cell cancer; Breast cancer; Rectal cancer; Ovarian cancer; Liver cancer; Lung Cancer; Cervical cancer; Thyroid cancer; Prostate cancer; Parotid gland cancer; Pancreatic cancer		Diffuse large B- cell lymphoma	30	39

The coexistence of lymphomas and HCC has been mostly reported in clinical patients infected with hepatitis virus [67, 68], while cohort studies and case–control studies have consistently reported that the increased risks of both HCC and lymphoma are associated with infection of HCV or HBV [69–72]. A Danish HCV cohort (DANVIR) cohort study of liver cancer and non-Hodgkin lymphoma was performed in HCV-infected patients [73]. The results indicated that the 10-year risks for HCC and NHL in HCV-infected patients were 1.0% [95% CI: 0.8–1.3%] and 0.1% (95% CI: 0.1–0.2%), respectively [73]. Compared to general population, the HCV infected patients had a 62.91-fold increased risk of HCC (95% CI: 28.99–136.52), a 29.97-fold increased risk of NHL during the first year of follow-up (95% CI: 6.08–147.84), but a 1.26-fold increased risk of NHL after the first year (95% CI: 0.36–4.41) [73]. A greater propensity has been found for patients with HCV infection to develop NHL, especially MZL and DLBCL, but not FL or T cell lymphomas [74]. An etiopathological role of HCV in lymphoproliferative disorder has been previously described in several studies by the supportive data of the HCV-associated NHL in response to interferon (IFN) and ribavirin therapy [75, 76]. The known major site of HCV replication is in the liver parenchyma, but HCV RNA has been also detected in the peripheral blood mononuclear cells (PBMCs) of infected individuals, including B cells and T cells [77, 78]. As regards HCV associated lymphomagenesis, experimental data support either a direct transformation mechanism or an indirect transformation mechanism. The receptors including CD81, the scavenger receptor SR‐BI, and tight‐junction protein Claudin‐1 have been found to mediate directly HCV entry into cells [79–81]. Although an in vitro study was failed to demonstrate that HCV can infect PBMCs, the possibility still remains for HCV‐specific B cells to capture HCV RNA from circulation via anti‐HCV antibodies and rheumatoid factor [82, 83]. HCV entry and active HCV replication in PBMCs was supported by the study in which nearly half of HCV-positive mixed cryoglobulinemia (MC) patients (versus individuals without MC) was detected the HCV minus-strand RNA which required the cellular machinery to synthesize from the positive strand of viral DNA [84]. When lymphocytes infected with HCV, the HCV core protein (C) and non-structural protein 3 (NS3) could induce nitric oxide synthase (NOS) which led to somatic mutations of both oncogenes and tumor suppressor genes contributing to lymphomagenesis [85, 86]. On the other hand, direct HCV infection in lymphocytes might not be necessary because HCV could also serve as an indirect transformation agent to induce somatic mutations of both oncogenes and tumor suppressor genes. For example, an antigenic selection driven maturation process had been suggested for the underlying lymphomagenesis in MC and HCV-associated NHL patients [87, 88]. The HCV envelope glycoprotein E2 would not require direct infection of B cells by HCV because E2 is only expressed on the virion surface. This glycoprotein E2 could bind to B cells via CD81 which is associated with CD19 and CD21 to form a complex, while the CD81-CD19-CD21 complex signaling and B-cell receptor cross-activation were thought to promote B-cell proliferation and NHL development [89, 90].

**Fig. 2:**
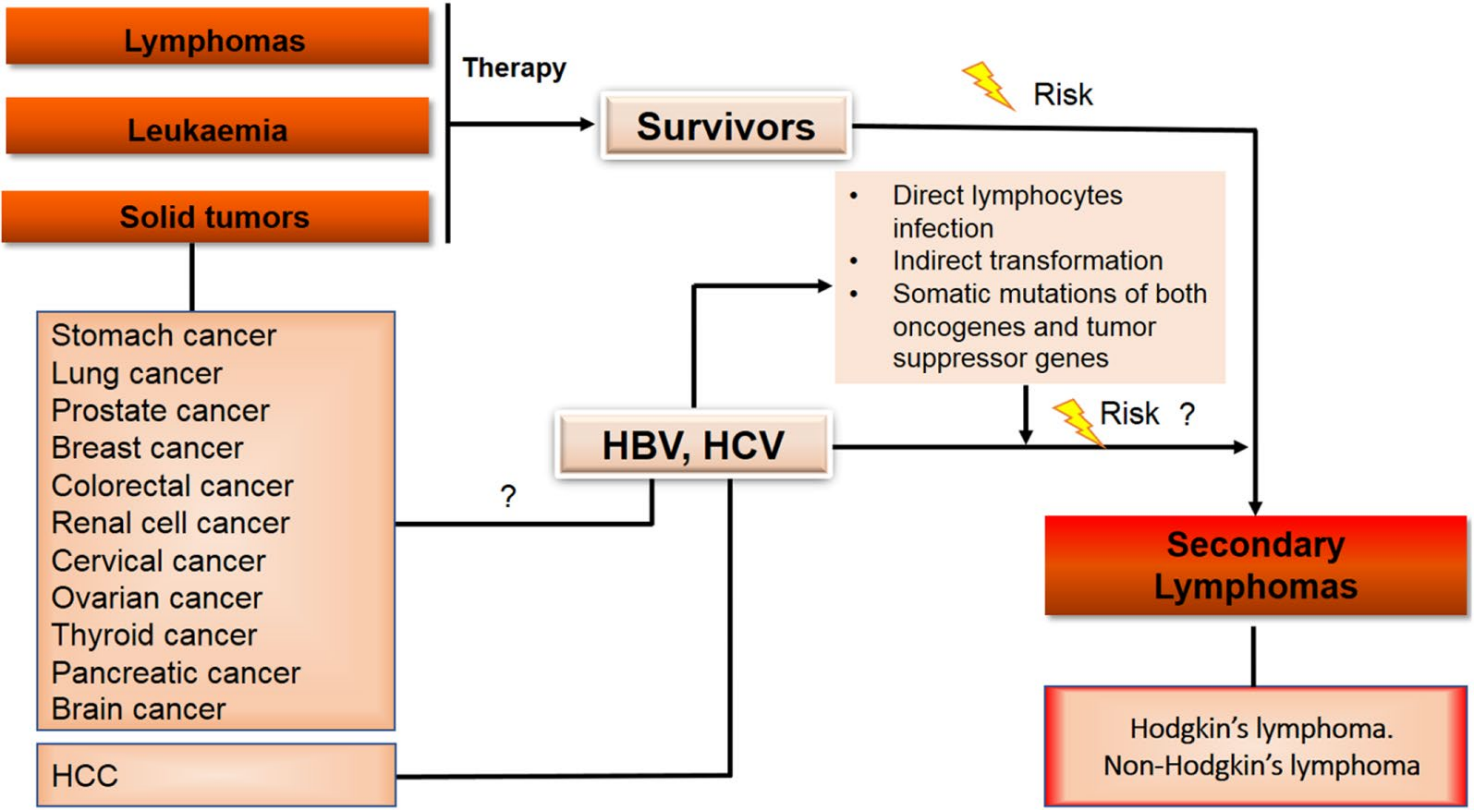
The schematic diagram for risk of secondary lymphomas in primary malignancies and etiologic risk factors of HCV and HBV

Contrary to HCV, the HBV infection in lymphoma patients was investigated less intensively even though the association between HBV infection and chronic lymphoproliferative disorders was reported in 1977 [91] much earlier than the reports on HCV in NHL patients. In respect to an etiologic role of HBV in lymphoma, there was a large number of experimental findings indicating that HBV could infect and replicate in the lymphoid cells [92]. Indeed, extrahepatic sites of HBV nucleic acids had been found in lymph nodes, spleen, gonads, thyroid gland, kidneys, pancreas, and adrenal glands from patients with acute HBV infection [93]. Nevertheless, the clinical implication of HBV activity in lymphomagenesis remained a matter of debate. Previously, it was reported that HBV-infected patients showed a 2–threefold higher risk to develop NHL, compared to non-infected patients [94]. However, a cohort study of patients infected with HBV from the Danish Cancer Registry a 17-fold higher risk of HCC for HBV-infected individuals but the risk of NHL was not higher in the HBV-infected cohort compared to non-HBV infected [72]. In a recent study, a cohort of seventy‐two patients with current or resolved HBV infection and B‐NHL between 2000 and 2017 were evaluated for the serological indicators of HBV activity [95]. The results indicated that the serological viral activity of HBV was significantly higher in indolent B‐NHL than aggressive B‐NHL [95]. The secondary lymphomas in primary malignancies and etiologic risk factors are summarized in Fig. 2.

## Lymphoma and metabolic disorders/diseases

Excess weight and obesity have been linked to many types of cancers including lymphoid neoplasms [96]. Previous meta-analyses show that a greater body mass index (BMI) may increase the risk of HL and NHL [97–100]. Latterly, additional prospective studies with a large number of cases have been published. In a systematic review and meta-analysis with over 20,000 NHL cases, anthropometric factors are correlated to the NHL risk, in which chronic lymphocytic leukemia/small lymphocytic lymphoma is positively associated with BMI in early adulthood and with height, whereas follicular lymphoma (FL) was only positively associated with height. The increased NHL risk with taller stature may reflect cumulative exposure to hormones/growth factors and nutrition status in early life but many other potential confounders need to be clarified [101]. An updated systematic review of published prospective studies is reported recently. The summary relative risk (RR) per 5 kg/m^2^ increase in BMI were 1.12 [95% confidence interval (CI): 1.05–1.20] for HL, 1.05 (95% CI: 1.03–1.08) for NHL, 1.11 (95% CI: 1.05–1.16) for DLBCL, respectively [102]. The waist-to-hip ratio was associated with an increased risk of DLBCL by 12% [102]. Although the pathophysiological and biological mechanisms linking obesity and risk of lymphoma are largely unknown, the most studied candidate systems include (i) adipocytokines, (ii) insulin/insulin-like growth factors (IGFs), and (iii) inflammation. It has been found that body fat can lead to changes in circulating levels of adipocytokines such as adiponectin and leptin, which can affect insulin resistance and inflammation [103–105]. Obesity may increase the risk of lymphoma by affecting insulin resistance and hyper-insulinemia which leads to increased bio-available insulin-like growth factor-I (IGF-I), a well-known growth factor that promotes cell proliferation and inhibits apoptosis [103].

Diabetes mellitus (DM) is a common metabolic disease and is predicted to be one of the five leading disease-burden contributors by 2030 [106]. There is increasing evidence that insulin and IGF-1 are involved in cellular events such as proliferation and metastasis, suggesting that DM is associated with the development and progression of malignancy [107]. Several previous meta-analyses have been performed previously to investigate the association between DM and NHL risk [108–110], and a positive association between DM and risk of NHL is suggested. However, the results from previous studies remain inconsistent because of methodological limitations in terms of the included case–control studies. A recent meta-analysis of 35 cohort studies demonstrated that the association between DM and NHL was much more substantial in an Asian population, while sensitivity analyses suggested the robustness of a positive association between DM and NHL risk [111]. Studies also reported the effect of metformin, one of the most commonly used medications for the treatment of type 2 DM (T2DM), on the outcome in T2DM patients suffering from lymphoma. In a study with a small sample size using the Computerized Patient Record System at a Veterans Affairs Medical Center, significantly greater long-term survival was found in the metformin group (18 patients) than the non-metformin group (20 patients) in lymphoma (5.89 vs 1.29 years, P < 0.001)[112]. In a large cohort study of 610,089 newly diagnosed T2DM patients with 2 or more times of prescription of antidiabetic drugs during 1999–2009, the NHL incidence was followed up until the end of 2011. The results indicated that the use of metformin was associated with a lower risk of NHL compared with non-metformin antidiabetic drugs [113]. Although some reports indicated that metformin might improve outcomes of lymphoma patients, the results from the previous studies showed inconsistent. In a cohort study of newly diagnosed DLBCL (n = 869) and FL (n = 895) patients enrolled in the Mayo component of the Molecular Epidemiology Resource cohort study between 2002 and 2015, the results showed that use of metformin was not associated with improved outcomes in newly diagnosed DLBCL and FL [114]. Metformin has been repeatedly shown to regulate lipid metabolism, not only via its antidiabetic effect but also through the activation of adenosine monophosphate kinase (AMPK) which mediates the regulations of peroxisomal proliferator-activated receptor α (PPARα) and PPARγ [115–117]. Studies have shown that PPARα agonists such as fenofibrate and clofibrate significantly reduce cell viability and induce apoptosis in lymphoma cell lines [118, 119]. PPARγ activation contributes to the survival of T lymphoma cells by affecting cellular metabolism [120]. The use of PPARγ ligands could inhibit proliferation and induce apoptosis in MCL [121]. The PPARγ ligands-induced apoptosis was also found in B lymphocytes, Burkitt’s B cell lymphoma cells [122], and DLBCL cells through a PPARγ -independent pathway [123]. A study reported the inhibition of lymphoma cell proliferation by PPARγ ligands via wingless-related integration site (Wnt) signaling pathway [124], which was well accepted as a potential mechanism contributing to lymphomagenesis. Apoptosis induction by PPARγ ligands is also considered for the importance of immune activity, e.g., a study has shown that engagement of CD40 to deliver a potent prosurvival signal prevents the PPARγ agonist-induced apoptosis of B lymphocytes via an NF-kappaB-dependent mechanism [125].

A study, based on multiple pathway crosstalk networks (PCNs), was performed recently to investigate the potential pathways in primary mediastinal B-cell lymphoma (PMBL) via analyzing the data of gene expression, pathway, and protein–protein interaction [126]. In this study, nonalcoholic fatty liver disease (NAFLD) was identified as a pathological mechanism of PMBL and to be one of the most important five hub pathways [including NAFLD, tuberculosis, human T-lymphotropic virus type-I (HTLV-I) infection, hepatitis B, and Epstein-Barr virus infection][126]. Nonalcoholic steatohepatitis (NASH), defined as the liver manifestation of metabolic syndrome and chronic inflammation, is the most severe form of NAFLD [127]. Evidence indicates that NASH patients are at high risk for HCC as well as a variety of other cancers [128]. In fact, liver involvement is not a rare condition in patients with lymphoma including HL, DLBCL, BL, T-cell lymphomas, and marginal zone B-cell lymphomas (MZL)[129]. Therefore, NASH characterized by chronic liver inflammation may represent a possible mechanistic link with lymphomagenesis which deserved further investigation. Although primary hepatic lymphoma (PHL) is a rare clinical entity comprising 0.016% of all cases of NHL and 0.4% of extranodal NHL [130], a substantial number of primary hepatic mucosa-associated lymphoid tissue (MALT) lymphomas were reported to occur in patients with chronic hepatitis or with primary biliary cirrhosis [131–133], suggesting a link between chronic liver inflammation and hepatic MALT lymphomagenesis. A case of hepatic MALT lymphomas with NASH diagnosis was reported [134]. On the other hand, the cases were reported in which NASH occurred in the patient with T-lymphoblastic lymphoma during chemotherapy including prednisolone [135] and fatty liver developed in children with non-Hodgkin lymphoma [136]. NASH was induced by induction chemotherapy for pediatric acute lymphoblastic leukemia [137], and even fulminant hepatic failure developed in a woman diagnosed with stage IV Hodgkin’s disease with a short course of prednisone (2 weeks) treatment while post-mortem demonstrated NASH and trivial liver involvement of lymphoma [138]. In a study involving 227 lymphoma patients to follow steatosis using a cut-off value of 42 Hounsfield units on the unenhanced CT part of PET/CT examinations, hepatic steatosis was observed in 11.9% of the patients at some point during their baseline or post-treatment evaluation [139].

**Fig. 3:**
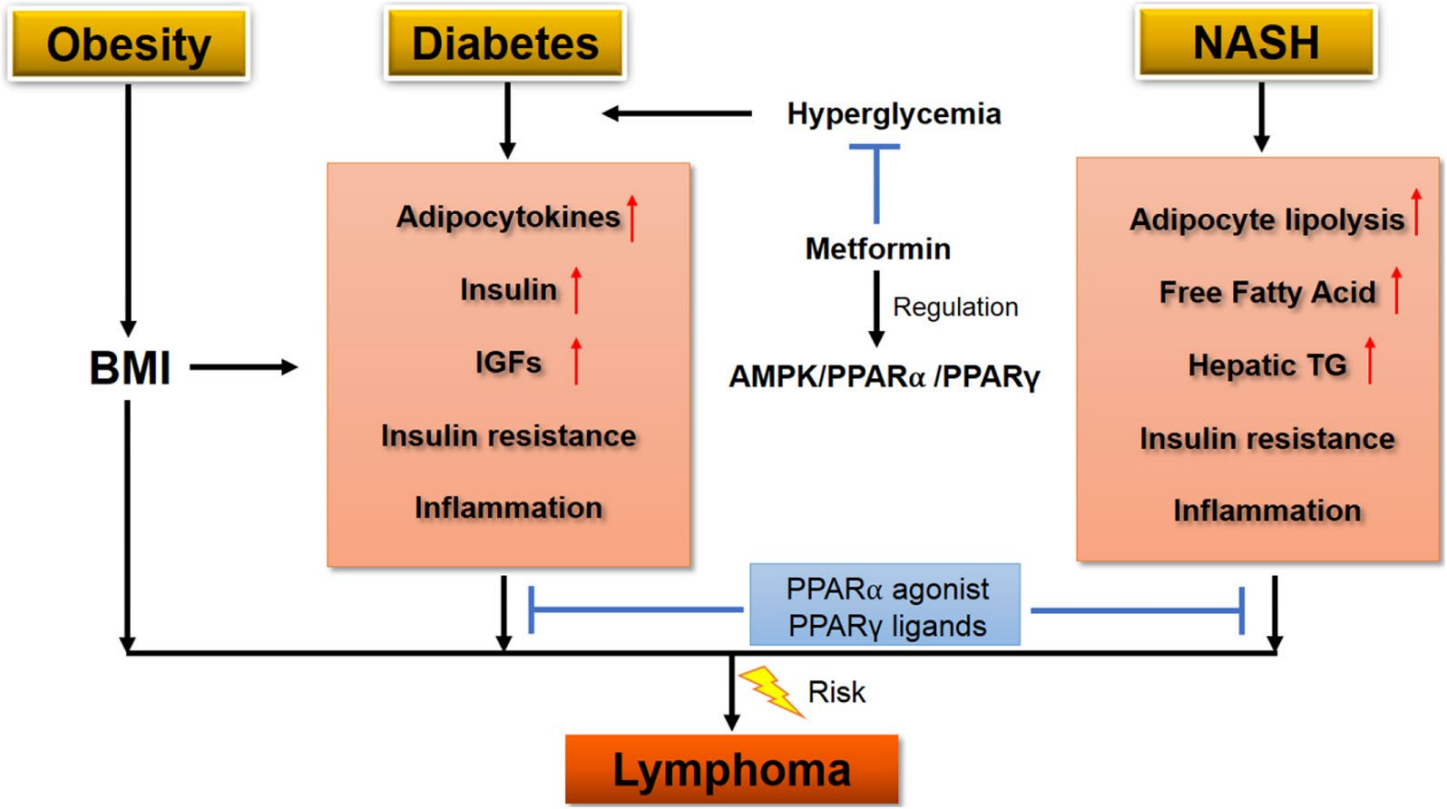
Schematic diagram of metabolic diseases and associated signaling transductions related to the risk of lymphomas

The metabolic diseases and associated signaling transductions related to the risk of lymphomas are summarized in Fig. 3.

Obese, diabetes and NASH can lead to metabolic abnormalities of adipose tissue, affecting the release of hormones, adipokines, inflammatory cytokines, growth factors, and free fatty acids [140]. All these metabolic substrates have been implicated as risk factors for lymphomas [141, 142]. Metabolic abnormalities of adipose tissue are associated with chronic inflammation which promotes the production of proinflammatory factors, such as TNF-α, IL-6, and IL-8, and inhibits the secretion of anti-inflammatory factors, such as adiponectin [143, 144]. Adiponectin, an adipose tissue specific cytokine that has a protective role against metabolic disturbances in diabetes and obesity, have been reported to suppress lymphoma growth in mice by modulating NK cells, CD8 T cells, and myeloid-derived suppressor cells [145]. Rimming of adipocytes by lymphoma cells has been recognized in several different entities of lymphoma with skin involvement [146]. The interaction between obese adipocytes and cancer cells can leads to transformation of adipocytes into cancer-associated adipocytes (CAAs) which may promote lymphoma via secreting more leptin and reducing the production of adiponectin [147]. In NASH, chronic liver inflammation causes lipolysis and excessive free fatty acids (FFAs) which are associated with hepatic infiltration of T helper 17 (Th17) cells [148] which mediate the progression from NASH to HCC [149, 150]. Besides its established role of NASH-HCC transition [151], NASH could act as a possible inflammatory trigger of lymphomagenesis, e.g., the breast implant-associated anaplastic large cell lymphoma cell lines and clinical specimens reveal a prominent Th1/Th17 phenotype in advanced disease [152]. In addition, reprogramming of fatty acid metabolism in NASH condition could also contribute to lymphomagenesis. For examples, upregulation of fatty acid synthase (FASN) and de novo lipogenesis are found to associate with the development to a more aggressive phenotype of NHL [153, 154] while inhibition of FASN can induced apoptosis in lymphoma cells and improving antitumor efficacy of chemotherapy [155, 156]. Although evidence suggests a close relationship between metabolic abnormalities and development of lymphomas, it is largely unknown about the lymphomagenesis in obesity, diabetes and NASH. Many important questions remain regarding the microenvironment of metabolic disorder in promoting lymphoma development and progression. Therefore, a more in-depth understanding is required to study, (1) the adipocytes and immune cells in metabolic tumor microenvironment interacting with lymphoma cells and contributing to lymphoma development and progression; (2) identification of specific molecular targets in metabolic pathways to limit tumor proliferation; and (3) determination of new strategies to block the crosstalk between lymphoma cells and tumor associated cells in metabolic tumor microenvironment.

In summary, SMNs remain critical research fields with regard to the etiopathogenesis, carcinogenesis, and optimal management. Therefore, the special emphasis is given to the following important areas. First, it is largely unknown for the genetic factors of SMNs in lymphomas, further study is needed to investigate, in particularly, the heritable diseases and associated genes to elucidate the potential mechanism(s) of SMN carcinogenesis in lymphomans. Second, although large-scale study is important to reveal the potential environmental epidemiology, it is needed to establish the experimental models to elucidate the molecular mechanisms of chemical exposures on the lymphomagenesis-associated SMNs. Third, the late effects of therapy remain the most important issue need to be solved because appropriate clinical manipulation for cancer survivors can prevent or even reverse the carcinogenic process. Multiple variable parameters such as patient height, weight, and size, volume and shape of the tumor should be considered to decide the irradiation/chemo modalities. Last, evidence suggests a close relationship between metabolic abnormalities and development of lymphomas, however it is largely unknown about the lymphomagenesis in the disease conditions such as obesity, diabetes and NASH. More studies are needed to gain insight into the lymphomagenesis linking to chronic inflammation and lipid metabolism which affect inflammatory cytokines, release of hormones, growth factors, adipokines, and free fatty acids. Moreover, as the oncology community applies more cutting-edge technologies such RNA sequencing molecular tools to reveal potential diagnostic and therapeutic targets, a systematic approach to the collection of data on SMNs should be incorporated into the prospective cohort studies of cancer patients.

## Data Availability

N/A.
